# Prognostic analysis of first-recurrence/metastasis esophageal squamous cell carcinoma: chemoimmunotherapy combined with radiotherapy as one of the optimal clinical management strategies

**DOI:** 10.3389/fimmu.2026.1791354

**Published:** 2026-07-21

**Authors:** Yitong Li, Qiaofang Li, Kuo Wang, Mengchang Gao, Lei Tian, Miaomiao Liu, Yuqing Bu, Yujie Cui

**Affiliations:** 1 The sixth Oncology Department, HeBei General Hospital, Shijiazhuang, Hebei, China; 2 The Fifth Oncology Department, HeBei General Hospital, Shijiazhuang, Hebei, China; 3 HeBei Sub bureau of North China Regional Air Traffic Management Bureau, Shijiazhuang, Hebei, China

**Keywords:** chemotherapy, distant metastatic esophageal cancer, esophageal squamous cell carcinoma, immunotherapy, Inverse Probability of Treatment Weighting, radiotherapy

## Abstract

**Background:**

The real-world efficacy and prognostic factors of First-line and subsequent-line treatment for first-recurrent/metastatic esophageal squamous cell cancer (ESCC) have not been fully evaluated.

**Methods:**

This single-institutional retrospective cohort study evaluated 156 first-recurrent/metastatic ESCC patients treated with First-line and subsequent-line treatment between January 2018 to January 2023. We assessed tumor response, long-term survival, and prognostic factors.

**Results:**

Among 156 ESCC patients with measurable lesions, in the first-line treatment setting, the combined Immunotherapy+Radiotherapy+Chemotherapy (IT+RT+CT) group had a lower incidence of progressive disease (PD) and Death compared with the Chemotherapy+Immunotherapy (CT+IT) group (80.0% vs 90.6%). First-Line Treatment were independent factors that affect progression-free survival (PFS), better PFS in the IT+RT+CT group compared to the CT+IT group (HR: 0.50, 95%CI: 0.28-0.90, P = 0.020). In the First-line and subsequent-line treatments, IT+RT+CT were identified as significant prognostic factors of overall survival (OS) through Cox proportional hazards analysis. After inverse probability of treatment weighting (IPTW) between the IT+RT+CT group and the CT+IT group in the First-line and Subsequent-line Treatments, the 1-, 2-year OS were 76.2% and 46.5% in the IT+RT+CT group, and 1-, 2-year OS were 39.5% and 19.4% in the CT+IT group, respectively (P = 0.011). In the doubly robust Cox proportional hazards model, which incorporated IPTW with weight trimming and adjustment for residual confounders (SMD>0.1), in the First-line and Subsequent-line Treatments, the IT+RT+CT regimen was significantly associated with improved OS compared to the CT+IT regimen (HR: 0.48, 95%CI: 0.27-0.85, P = 0.011).

**Conclusions:**

Our study demonstrated that, IT+RT+CT as first-line treatment for recurrent/metastatic ESCC significantly prolonged PFS compared to CT+IT. Furthermore, IT+RT+CT extended OS in First-line and subsequent-line treatments. However, these findings require validation in prospective studies to confirm their clinical significance.

## 1 Introduction

Esophageal cancer (EC) is the seventh most common cancer and the sixth leading cause of cancer-related mortality (1). The 5-year survival rate for all stages of EC is approximately 20% in both China and the United States, while it is only 12% in Europe (2, 3). Esophageal squamous cell carcinoma (ESCC), which predominantly occurs in the upper and middle esophagus, represents the major EC subtype in Asia and Eastern Europe. Conventional treatment options for EC patients include surgery, chemotherapy, chemoradiotherapy, and targeted therapy (4). Esophageal cancer remains prone to locoregional recurrence and distant metastasis despite multimodal treatments, constituting the leading cause of mortality in affected patients (5, 6). Despite curative-intent esophagectomy, approximately 50% of patients with esophageal cancer will experience disease recurrence (7). The prognosis for recurrent disease remains dismal, characterized by significantly limited survival outcomes and scarce prospects for durable remission (8, 9). Regional recurrence likely indicates non-widespread metastatic disease, making it potentially suitable for more intensive treatment (7). For locally recurrent disease, repeat local therapy (surgery or radiation) with curative intent may represent a therapeutic option (9). The 5-year overall survival rate for metastatic esophageal cancer is consistently reported to be below 25% in clinical studies (10). Oligometastatic esophageal cancer (OMEC), as an intermediate state between locally advanced and widely metastatic disease, has a better prognosis compared to polymetastatic esophageal cancer and may benefit from local radiotherapy (11). Systemic treatments (such as chemotherapy) also play a certain role in recurrent and metastatic esophageal cancer, but their efficacy is limited (9). Due to the limitations of conventional treatment methods, researchers have been dedicated to developing novel and effective therapies in recent years, including immunotherapy (such as PD-1 inhibitors) (12). The treatment of recurrent and metastatic esophageal cancer continues to pose significant challenges, necessitating further research to improve patient outcomes. Current therapeutic strategies should be individualized based on recurrence patterns and patient status, while actively exploring novel treatment approaches. Although recent clinical trials have demonstrated improved survival in patients with metastatic esophageal cancer, the number of participants in these trials remains limited. Moreover, the survival benefits observed in clinical trials may not be generalizable to the broader patient population. Pape M et al. (13) selected patients with metastatic esophageal cancer diagnosed between 2006 and 2020 from the nationwide Netherlands Cancer Registry. Survival was calculated for different percentiles of the survival curve for each incidence year. The results showed that the median overall survival of esophageal cancer patients showed no significant change (n=10,448; from 5.2 months to 5.2 months, P = 0.06). However, the best-case scenario survival for esophageal cancer patients significantly improved from 17.2 months to 21.0 months (P = 0.006). Most patients in routine clinical practice did not show survival improvements. Therefore, this study analyzed the data of ESCC patients with first-recurrence/metastasis in the real-world database, analyzed their independent prognostic factors, and explored the important prognostic factors affecting this patient population. These findings provide evidence for decision-making in clinical practice and provide more reference data for the design of future clinical trials.

## 2 Materials and methods

### 2.1 Patients

This is a retrospective review based on ESCC patients from the Hebei General Hospital from January 2018 to January 2023. Inclusion criteria included aged between 18 and 90 years old; confirmed by pathology with ESCC; staged with IVB stage ESCC (AJCC 8th); first recurrence or metastasis after initial treatment in patients with I-IVA stage ESCC (AJCC 8th); the performance status (PS) of the Eastern Cooperative Oncology Group (ECOG) that was 1 to 2; and with normal hematologic, hepatic, and renal function. Exclusion criteria included patients with dead or Stable Disease (SD) after initial treatment in patients with Stage I-IVA stage ESCC (AJCC 8th). Additionally, patients needed to have at least one measurable or evaluable lesion, as defined in the Response Evaluation Criteria in Solid Tumors (RECIST) guidelines (version 1.1). This study was approved by the Hebei General Hospital Ethics Committee (Approval ID: 2025-LW-0286). This study complies with the Declaration of Helsinki.

### 2.2 Data collection

Clinical information included age, sex, the clinical characteristics before initial recurrence and metastasis (including histological grade, primary tumor location, TNM stage, tumor length, and tumor site), Prior treatment (which outlined the radical treatment strategies for I-IVA stage ESCC prior to disease progression, including Prior Radiotherapy, Prior Operation, Prior Chemotherapy, Prior Immunotherapy), Progression After Prior Treatment (which refers to the first pattern of recurrence/metastasis in patients with stage I-IVA disease following radical treatment, including Local Progression After Prior Treatment, Regional Progression After Prior Treatment, and Distant Progression After Prior Treatment), Organ Metastasis (which included patients with stage I-IVA disease who develop visceral metastases after their first progression, as well as patients with stage IVB disease who present with visceral metastases at diagnosis), First Line Treatment, First Line And Subsequent Line Treatments.

### 2.3 Treatment

This is a single-arm, retrospective study conducted at Hebei General Hospital in China, where patients received treatment approaches encompassing Radiotherapy (RT), Chemotherapy (CT), immunotherapy (IT), and surgical intervention. The RT regimen encompasses three primary types: Definitive radiotherapy: 58–66 Gy in 25–33 fractions, Postoperative adjuvant radiotherapy: 45–54 Gy in 25–27 fractions, Radiotherapy for locoregional recurrence: 50–66 Gy in 25–33 fractions. All RT protocols involve irradiation of the esophagus and its lymphatic drainage areas, with potential inclusion of non-regional lymph nodes (e.g., supraclavicular, retroperitoneal, para-aortic lymph nodes). The decision to administer radiotherapy for distant organ metastases is determined by the attending physician, based on the patient’s physical condition and personal preferences.

Patients received platinum-based chemotherapy administered every three weeks, continuing for 4–6 cycles or until disease progression or the occurrence of intolerable toxic side effects. The specific treatment protocol was determined by the attending physician. First-line therapy: Paclitaxel combined with platinum or fluorouracil combined with platinum regimens. Subsequent-line therapy: Dual-agent or monotherapy, tailored to the patient’s physical condition.

Immunotherapy (IT) for esophageal cancer encompasses neoadjuvant, adjuvant, first-line, and later-line treatment strategies. PD-1/PD-L1 inhibitors primarily included sintilimab, camrelizumab, tislelizumab, and pembrolizumab, administered every three weeks until disease progression or the occurrence of an intolerable toxic reaction, with a duration of one to two years. Immune maintenance therapy refers to receiving more than six cycles of immunotherapy.

### 2.4 Statistical methods

Data processing and analysis were performed using SPSS soft-ware version 26.0 (lBM SPSS) and R version 4.5.0. The chi-squared test (or Fisher’s exact test, if appropriate) was used to analyze the differences between patients grouped by categorical variables. Inverse Probability of Treatment Weighting (IPTW) targeting the Average Treatment Effect on the Treated (ATT) was performed to adjust for baseline differences between patient groups. Treatment group retained a fixed weight of 1, while control group were weighted to match the treated group’s covariates. We assessed the balance of covariates using Standardized Mean Differences (SMD), with an SMD < 0.1 indicating adequate balance. The effective sample size (ESS) was monitored to evaluate the precision of the weighted estimates. To address potential instability due to extreme weights, we applied weight trimming at the 1st and 99th percentiles. Finally, weighted Cox proportional hazards models were fitted using the survey package to estimate the hazard ratios (HRs) and 95% confidence intervals (CIs) for the association between treatment groups. To validate the robustness of findings, we performed two complementary adjusted survival analyses. The conventional IPT-weighted Kaplan-Meier approach, which directly reweights the risk sets using the ATT-targeted IPTW weights, without incorporating an additional outcome regression model. The primary doubly robust IPTW-Cox framework, which combines IPTW with a weighted Cox model via G-computation, provides unbiased estimates if either the weighting or outcome model is correctly specified.

OS is defined as the time from the first occurrence of recurrence or metastasis after initial treatment in patients with Stage I-IVA disease to death from any cause. For Stage IVB patients, OS it is defined as the time from diagnosis to death from any cause. If a patient remains alive at the end of the study, the time of the last follow-up is used as the endpoint. The PFS for patients with Stage I-IVA disease is defined as the time from the first occurrence of recurrence/metastasis after initial treatment to the time of tumor progression or death from any cause. For patients with Stage IVB disease, PFS is defined as the time from the start of first-line treatment to tumor progression or death from any cause. If a patient remains alive without disease progression at the end of the study, the date of the last follow-up is used as the endpoint. Survival curves were generated using Kaplan-Meier methods, and compared by log-rank test. Univariate and multivariate Cox regression analyses were used to analyze the independent risk factors for PFS and OS. Only variables with statistical significance (P<0.05) in univariate analysis were incorporated into multivariate analysis. Hazard ratios (HR) were calculated based on multivariable Cox proportional hazards models to estimate predictors of PFS and OS. All CIs were stated at the 95% confidence level. P<0.05 indicated a statistically significant difference.

## 3 Results

### 3.1 Clinical data and patient characteristics

According to the inclusion and exclusion criteria, a total of 156 ESCC patients with recurrent and/or distant stage were included. There was a higher percentage of I-IVA stage patients (I-III 46.15%;IVA 6.41%) who received prior treatment (prior Radiotherapy 23.08%, prior Operation 28.21%, prior Chemotherapy 39.10%, prior Immunotherapy 12.18%), progression after prior treatment (Regional 17.95%, Local 26.92%, Distant 26.92%). 74(47.44%) patients were IVB stage, with Organ Metastasis (27.56%). Most patients received first-line treatment with Chemotherapy combined PD-1/PD-L1 inhibitors (33.97%).The most common treatment modality in First-line and Subsequent-Line treatment is the combination of IT+RT+CT (40.38%).The clinical and pathological features were showed in **Table 1**.

**Table 1:** Clinicopathologic features of entire dataset.

Variables	Total (n = 156)
Sex	
female	61 (39.10)
male	95 (60.90)
Age	
<70 years	92 (58.97)
≥70 years	64 (41.03)
Histological grade	
G1	2 (1.28)
G2	38 (24.36)
G3	40 (25.64)
G0/x	76 (48.72)
TNM stage	
I-III	72 (46.15)
IVA	10 (6.41)
IVB	74 (47.44)
Tumor length	
<5cm	46 (29.49)
≥5cm	80 (51.28)
unknown	30 (19.23)
Tumor location	
Cervical+Upper thoracic	30 (19.23)
Middle thoracic	66 (42.31)
Lower thoracic	54 (34.62)
unknown	6 (3.85)
Prior radiotherapy	
no	120 (76.92)
yes	36 (23.08)
Prior operation	
no	112 (71.79)
yes	44 (28.21)
Prior chemotherapy	
no	95 (60.90)
yes	61 (39.10)
Prior immunotherapy	
no	137 (87.82)
yes	19 (12.18)
Regional progression after prior treatment	
no	128 (82.05)
yes	28 (17.95)
Local progression after prior treatment	
no	114 (73.08)
yes	42 (26.92)
Distant progression after prior treatment	
no	114 (73.08)
yes	42 (26.92)
Organ metastasis	
no	113 (72.44)
yes	43 (27.56)
First-line treatment	
CT+IT	53 (33.97)
IT+RT+CT	35 (22.44)
RT	19 (12.18)
RT+CT	49 (31.41)
First-line and subsequent-line treatments	
CT+IT	35 (22.44)
IT+RT+CT	63 (40.38)
RT	18 (11.54)
RT+CT	40 (25.64)

### 3.2 Prognostic factor analysis for 156 ESCC patients with recurrent and/or distant stage

**Figure 1:**
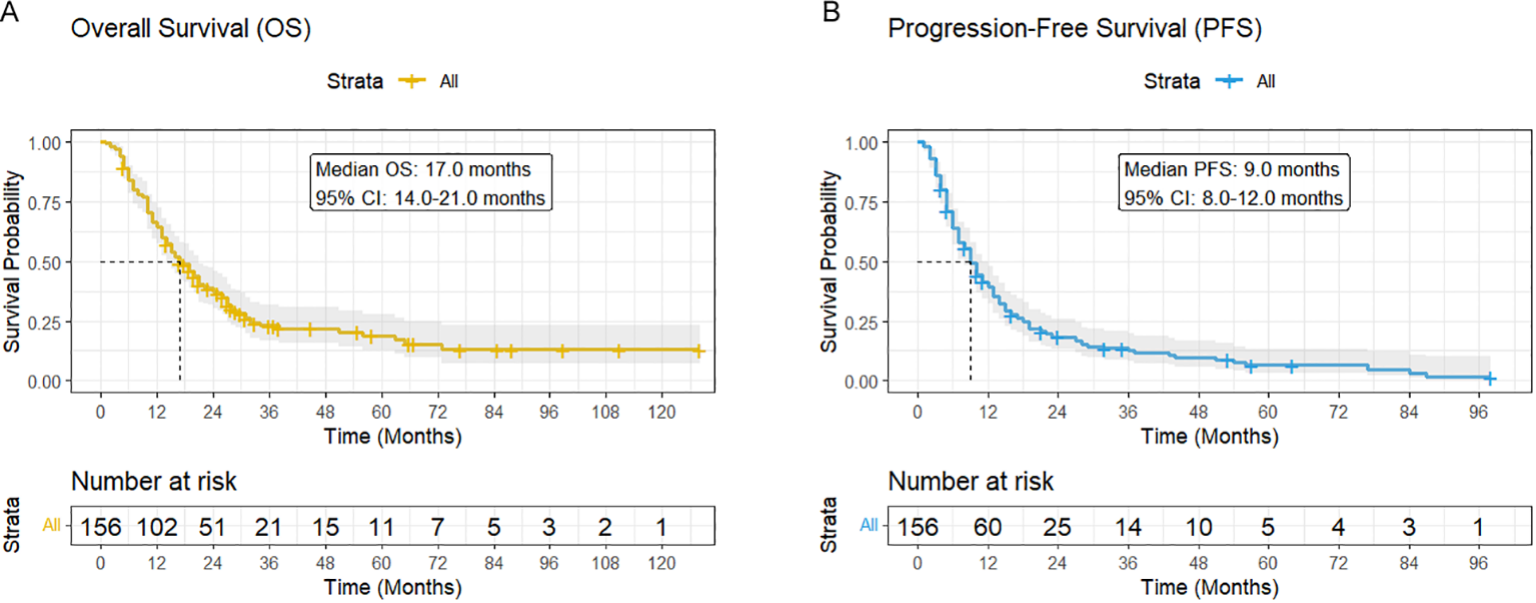
OS and PFS of 156 ESCC patients with recurrent and/or distant stage. **(A)** OS, **(B)**PFS.

In the entire database, there were 117 patient deaths, with a median follow-up of 38 months (IQR 28–77). The mOS was 17.0 months (95%CI:14.0-21.0) and the mPFS was 9.0 months (95%CI:8.0-12.0) (**Figure 1**), separately.

Univariate analysis revealed that Age, TNM stage, Tumor Length, Tumor Location, and First-line And Subsequent-line Treatments are prognostic factors that affect OS (**Table 2**). Tumor Length, Tumor Location, Organ Metastasis and First-Line Treatment are prognostic factors that affects PFS (**Table 2**).

**Table 2:** Univariate Cox regression analysis of 156 ESCC patients with recurrent and/or distant stage.

Variables	PFS		OS	
	HR (95%CI)	*P*	HR (95%CI)	*P*
Sex				
female	1.00 (Reference)		1.00 (Reference)	
male	1.14 (0.80 ~ 1.61)	0.460	1.13 (0.78 ~ 1.64)	0.523
Age				
<70 years	1.00 (Reference)		1.00 (Reference)	
≥70 years	1.22 (0.87 ~ 1.73)	0.252	1.48 (1.02 ~ 2.15)	**0.038**
Histological grade				
G1	1.00 (Reference)		1.00 (Reference)	
G2	1.21 (0.29 ~ 5.10)	0.794	0.71 (0.17 ~ 3.02)	0.644
G3	1.31 (0.31 ~ 5.49)	0.714	0.86 (0.20 ~ 3.61)	0.833
G0/x	1.38 (0.34 ~ 5.70)	0.655	0.99 (0.24 ~ 4.11)	0.990
TNM stage				
I-III	1.00 (Reference)		1.00 (Reference)	
IVA	1.98 (0.97 ~ 4.03)	**0.059**	3.06 (1.47 ~ 6.34)	**0.003**
IVB	1.03 (0.73 ~ 1.46)	**0.856**	1.33 (0.90 ~ 1.95)	**0.148**
Tumor length				
<5cm	1.00 (Reference)		1.00 (Reference)	
≥5cm	1.78 (1.19 ~ 2.68)	**0.005**	1.63 (1.05 ~ 2.51)	**0.028**
unknown	1.45 (0.88 ~ 2.38)	0.145	0.97 (0.55 ~ 1.71)	0.919
Tumor location				
Cervical+Upper thoracic	1.00 (Reference)		1.00 (Reference)	
Middle thoracic	1.67 (1.03 ~ 2.78)	**0.039**	1.28 (0.76 ~ 2.16)	0.352
Lower thoracic	2.08 (1.25 ~ 3.45)	**0.005**	1.72 (1.01 ~ 2.93)	**0.046**
unknown	2.03 (0.82 ~ 5.03)	0.125	1.51 (0.57 ~ 4.03)	0.410
Prior radiotherapy				
no	1.00 (Reference)		1.00 (Reference)	
yes	1.01 (0.68 ~ 1.50)	0.966	1.09 (0.71 ~ 1.68)	0.696
Prior operation				
no	1.00 (Reference)		1.00 (Reference)	
yes	0.92 (0.63 ~ 1.34)	0.676	0.70 (0.46 ~ 1.07)	0.104
Prior chemotherapy				
no	1.00 (Reference)		1.00 (Reference)	
yes	1.20 (0.86 ~ 1.68)	0.293	1.01 (0.69 ~ 1.46)	0.971
Prior immunotherapy				
no	1.00 (Reference)		1.00 (Reference)	
yes	1.22 (0.73 ~ 2.03)	0.456	1.11 (0.62 ~ 1.99)	0.734
Regional progression after prior treatment				
no	1.00 (Reference)		1.00 (Reference)	
yes	1.33 (0.87 ~ 2.03)	0.187	1.11 (0.70 ~ 1.77)	0.646
Local progression after prior treatment				
no	1.00 (Reference)		1.00 (Reference)	
yes	0.80 (0.55 ~ 1.17)	0.242	0.88 (0.58 ~ 1.33)	0.533
Distant Progression after prior treatment				
no	1.00 (Reference)		1.00 (Reference)	
yes	1.20 (0.82 ~ 1.75)	0.344	1.07 (0.71 ~ 1.62)	0.752
Organ metastasis				
no	1.00 (Reference)		1.00 (Reference)	
yes	1.50 (1.04 ~ 2.17)	**0.029**	1.30 (0.88 ~ 1.91)	0.192
First-line treatment				
CT+IT	1.00 (Reference)		1.00 (Reference)	
IT+RT+CT	0.57 (0.36 ~ 0.91)	**0.018**	0.62 (0.37 ~ 1.04)	0.069
RT	0.49 (0.27 ~ 0.88)	**0.016**	0.66 (0.36 ~ 1.23)	0.192
RT+CT	0.61 (0.41 ~ 0.92)	**0.019**	0.71 (0.46 ~ 1.09)	0.120
First-line and subsequent-line treatments				
CT+IT	1.00 (Reference)		1.00 (Reference)	
IT+RT+CT	1.00 (0.65 ~ 1.54)	0.997	0.58 (0.36 ~ 0.93)	**0.024**
RT	0.50 (0.27 ~ 0.95)	**0.033**	0.47 (0.23 ~ 0.95)	**0.035**
RT+CT	0.61 (0.38 ~ 0.99)	**0.048**	0.63 (0.37 ~ 1.06)	0.083

Bold values indicate statistically significant differences (P < 0.05).

**Figure 2:**
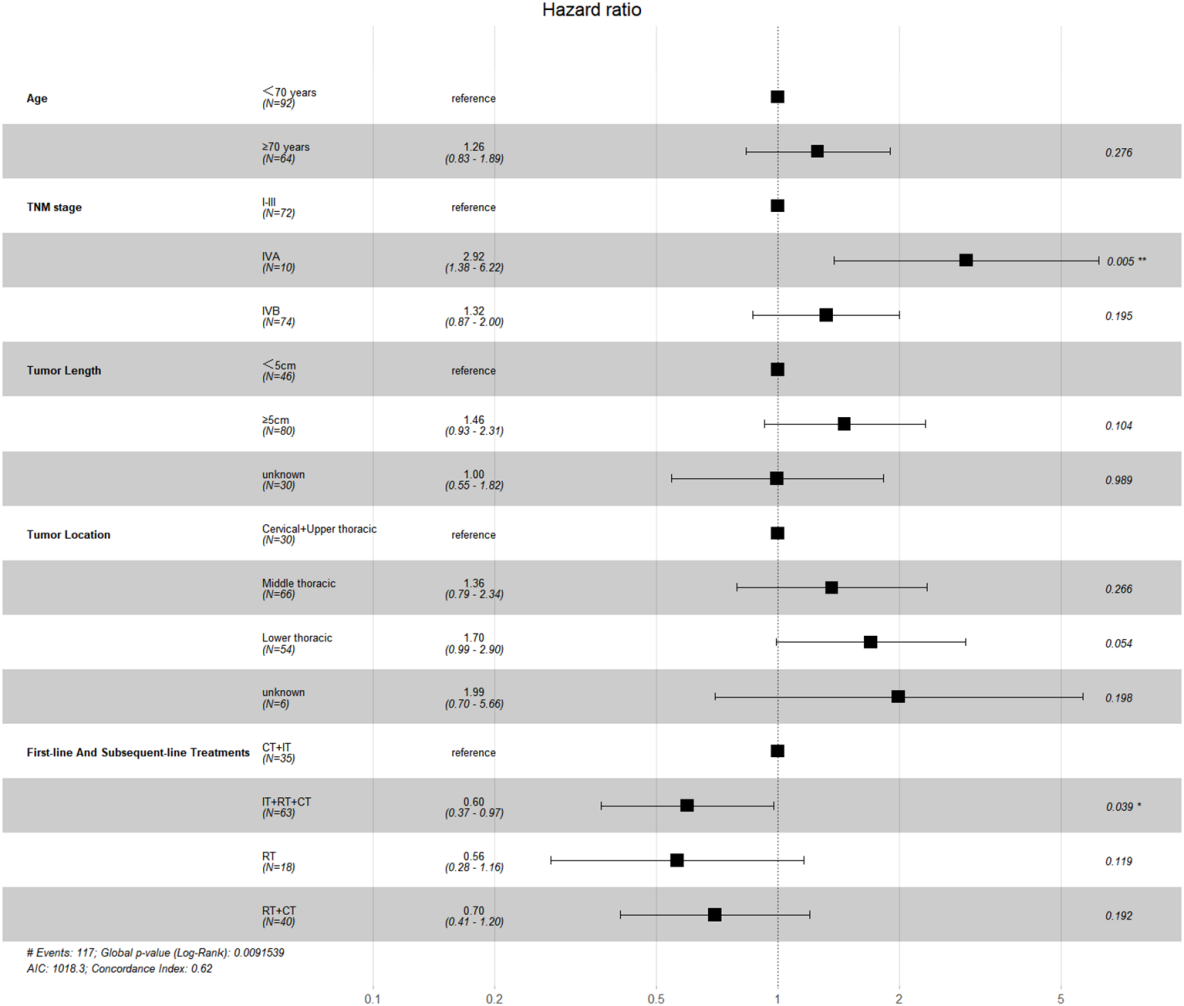
Multivariate Cox regression analysis for OS of 156 recurrent and/or distant ESCC patients. * *P* < 0.05, ** *P*< 0.01.

**Figure 3:**
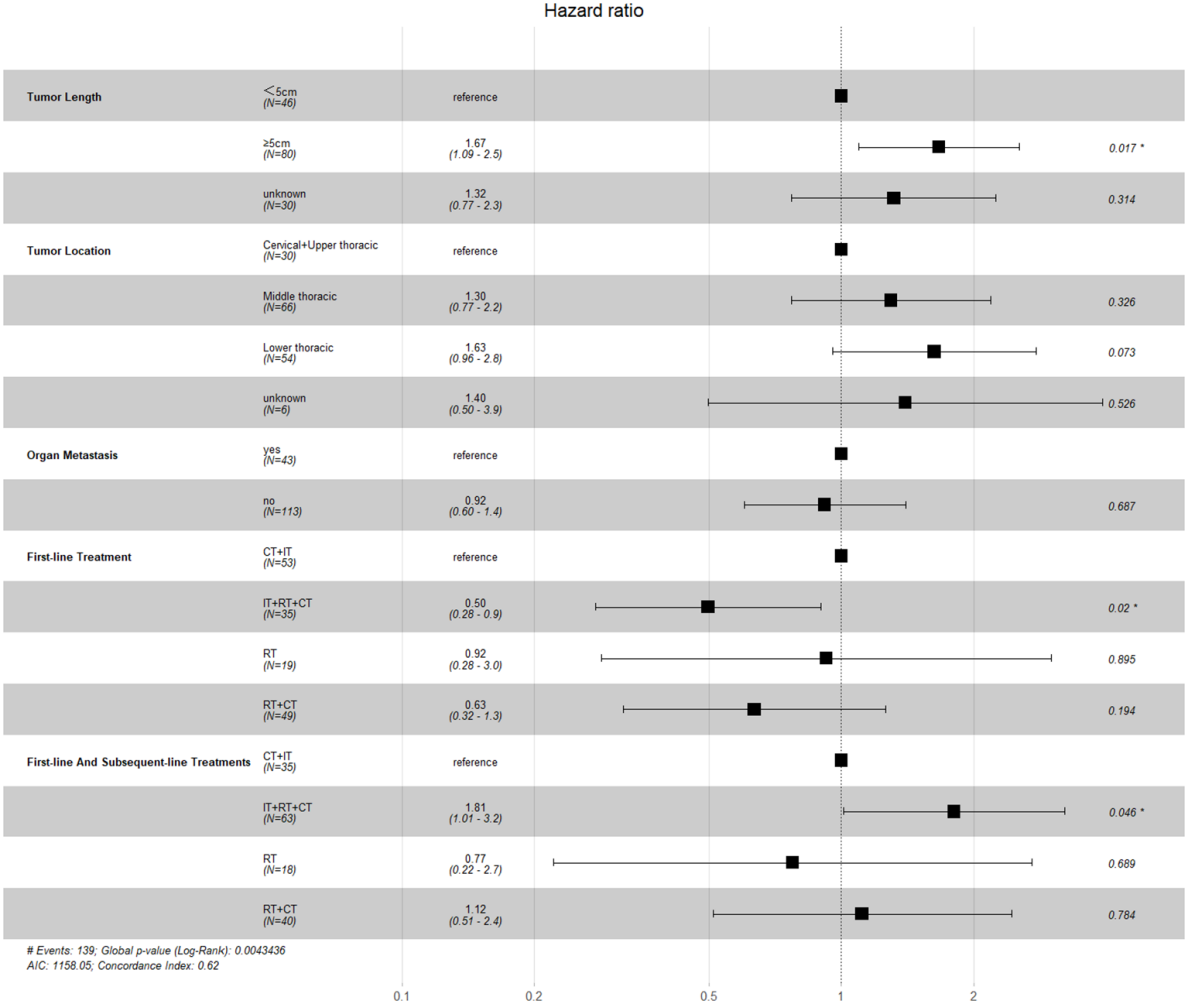
Multivariate Cox regression analysis for PFS of 156 recurrent and/or distant ESCC patients. * *P* < 0.05, ** *P*< 0.01.

Multivariate analysis revealed that TNM stage and First-line And Subsequent-line Treatments were independent factors for OS (**Figure 2**); Tumor length, First-Line Treatment and First-line And Subsequent-line Treatments were independent factors that affect PFS (**Figure 3**). In First-line treatment, there were better PFS of the patients of the IT+RT+CT group compared to the CT+IT group (HR: 0.50, 95%CI: 0.28-0.90, P = 0.020) (**Figure 3**). In First-line And Subsequent-line Treatments, there were better OS of the patients of the IT+RT+CT group compared to the CT+IT group (HR: 0.60, 95%CI: 0.37-0.97, P = 0.039) (**Figure 2**).

### 3.3 Failure pattern after first-line treatment for 156 recurrent and/or distant patients

The failure pattern after first-line treatment was analyzed. Among the entire cohort of 156 ESCC patients, 90 (57.7%) experienced progressive disease (PD), with 33 (62.3%) in the CT+IT group, 23 (65.8%) in the IT+RT+CT group, 9 (47.4%) in the RT group, and 25 (51.0%) in the RT+CT group. In addition, 43 (27.6%) patients died, and 23 (14.7%) had stable disease (SD). The incidence of the composite endpoint of PD or death was lower in the IT+RT+CT group than in the CT+IT group (80.0% vs 90.6%). However, no statistically significant difference was observed in the distribution of failure patterns among the treatment groups (P = 0.452) (**Table 3**).

**Table 3:** Failure pattern after first-line treatment for 156 recurrent and/or distant patients.

Variables	Total (n = 156) (n%)	CT+IT (n = 53) (n%)	IT+RT+CT (n = 35) (n%)	RT (n = 19) (n%)	RT+CT (n = 49) (n%)	P
Progression After First-Line Treatment	90 (57.69)	33 (62.26)	23 (65.71)	9 (47.37)	25 (51.02)	0.452*
Distant metastasis	33 (21.15)	10 (18.87)	8 (22.86)	3 (15.79)	12 (24.49)	
Local recurrence	19 (12.18)	7 (13.21)	6 (17.14)	2 (10.53)	4 (8.16)	
Regional recurrence	12 (7.69)	3 (5.66)	4 (11.43)	0 (0.00)	5 (10.20)	
Local recurrence+regional recurrence	5 (3.21)	2 (3.77)	0 (0.00)	2 (10.53)	1 (2.04)	
Local recurrence+distant metastasis	6 (3.85)	2 (3.77)	2 (5.71)	1 (5.26)	1 (2.04)	
Regional recurrence+distant metastasis	12 (7.69)	6 (11.32)	3 (8.57)	1 (5.26)	2 (4.08)	
Local+regional recurrence+distant metastasis	3 (1.92)	3 (5.66)	0 (0.00)	0 (0.00)	0 (0.00)	
Dead	43 (27.56)	15 (28.30)	5 (14.29)	5 (26.32)	18 (36.73)	
Stable Disease (SD)	23 (14.74)	5 (9.43)	7 (20.00)	5 (26.32)	6 (12.24)	

*: Simulated p-value.

### 3.4 Analysis between the IT+RT+CT group and the CT+IT group according to first-line and subsequent-line treatments groups

**Figure 4:**
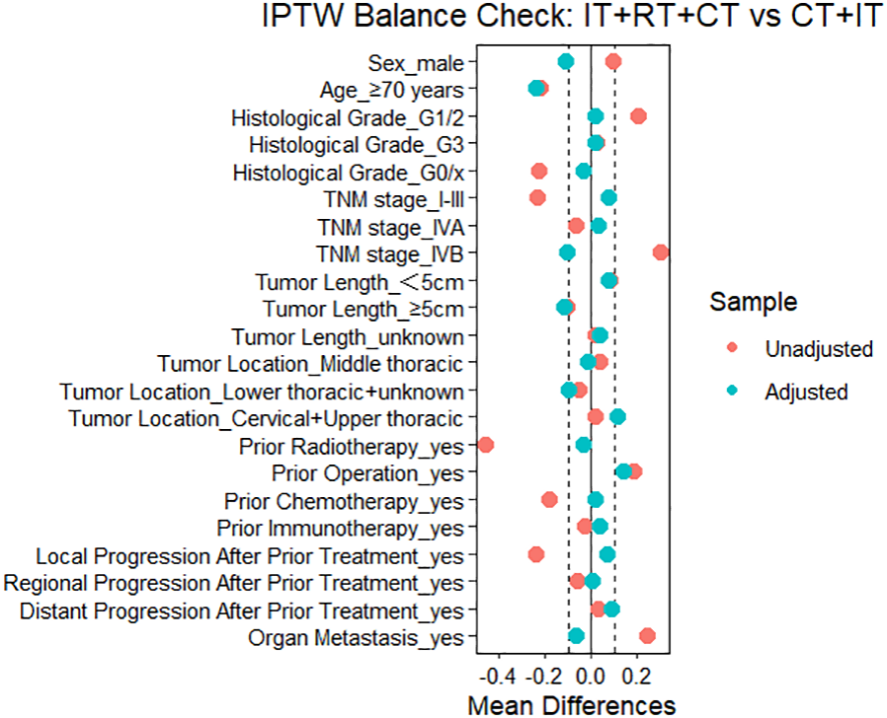
Clinical characteristics the IT+RT+CT group and CT+IT group in the first-line and subsequent-line treatments before and after IPTW .

Based on the results of multivariate analysis, the clinical data of the IT+RT+CT group and the CT+IT group in First-line and Subsequent-line Treatments were analyzed, revealing imbalance between the two groups (**Table 4**, **Figure 4**). After Inverse Probability of Treatment Weighting (IPTW) to adjust for confounders, the clinical characteristics between the two groups were successfully balanced (**Table 4**, **Figure 4**).

**Table 4:** Characteristics of patients before and after IPTW according to the IT+RT+CT group and CT+IT group in the first-line and subsequent-line treatments.

Variable	Before IPTW (n, %)			*P*	SMD	After IPTW (n%)		SMD
	Total (n = 98)	IT+RT+CT (n = 63)	CT+IT (n = 35)			IT+RT+CT	CT+IT	
Sex				0.349				
female	36 (36.73)	21 (33.33)	15 (42.86)		-0.095	33.33%	22.18%	0.112
male	62 (63.27)	42 (66.67)	20 (57.14)		0.095	66.67%	77.82%	-0.112
Age				**0.033**				
<70 years	56 (57.14)	41 (65.08)	15 (42.86)		0.222	65.08%	41.26%	0.238
≥70 years	42 (42.86)	22 (34.92)	20 (57.14)		-0.222	34.92%	58.74%	-0.238
Histological Grade				**0.046**				
G1/2	24 (24.49)	20 (31.75)	4 (11.43)		0.203	31.75%	30.09%	0.017
G3	24 (24.49)	16 (25.40)	8 (22.86)		0.025	25.40%	23.62%	0.018
G0/x	50 (51.02)	27 (42.86)	23 (65.71)		-0.229	42.86%	46.29%	-0.034
TNM stage				**0.010**				
I-III	44 (44.9)	23 (36.51)	21 (60.00)		-0.235	36.51%	29.03%	0.075
IVA	7 (7.14)	3 (4.76)	4 (11.43)		-0.067	4.76%	1.96%	0.028
IVB	47 (47.96)	37 (58.73)	10 (28.57)		0.302	58.73%	69.01%	-0.103
Tumor Length				0.568				
<5cm	25 (25.51)	18 (28.57)	7 (20.00)		0.086	28.57%	20.98%	0.076
≥5cm	55 (56.12)	33 (52.38)	22 (62.86)		-0.104	52.38%	64.00%	-0.116
unknown	18 (18.37)	12 (19.05)	6 (17.14)		0.019	19.05%	15.01%	0.040
Tumor Location				0.882				
Cervical + Upper thoracic	15 (15.31)	10 (15.87)	5 (14.29)		0.016	15.87%	4.27%	0.116
Middle thoracic	47 (47.96)	31 (49.21)	16 (45.71)		0.035	49.21%	50.90%	-0.099
Lower thoracic + unknown	36 (36.73)	22 (34.92)	14 (40.00)		-0.051	34.92%	44.83%	-0.017
Prior Radiotherapy				**<.001**				
no	71 (72.45)	56 (88.89)	15 (42.86)		0.460	88.89%	85.82%	0.031
yes	27 (27.55)	7 (11.11)	20 (57.14)		-0.460	11.11%	14.18%	-0.031
Prior Operation				**0.036**				
no	75 (76.53)	44 (69.84)	31 (88.57)		-0.187	69.84%	83.69%	-0.138
yes	23 (23.47)	19 (30.16)	4 (11.43)		0.187	30.16%	16.31%	0.138
Prior Chemotherapy				0.080				
no	59 (60.2)	42 (66.67)	17 (48.57)		0.181	66.67%	68.57%	-0.019
yes	39 (39.8)	21 (33.33)	18 (51.43)		-0.181	33.33%	31.43%	0.019
Prior Immunotherapy				0.707				
no	83 (84.69)	54 (85.71)	29 (82.86)		0.029	85.71%	89.44%	-0.037
yes	15 (15.31)	9 (14.29)	6 (17.14)		-0.029	14.29%	10.56%	0.037
Regional Progression After Prior Treatment				0.463				
no	82 (83.67)	54 (85.71)	28 (80.00)		0.057	85.71%	86.35%	-0.006
yes	16 (16.33)	9 (14.29)	7 (20.00)		-0.057	14.29%	13.65%	0.006
Local Progression After Prior Treatment				**0.011**				
no	71 (72.45)	51 (80.95)	20 (57.14)		0.238	80.95%	88.21%	-0.073
yes	27 (27.55)	12 (19.05)	15 (42.86)		-0.238	19.05%	11.79%	0.073
Distant Progression After Prior Treatment				0.762				
no	71 (72.45)	45 (71.43)	26 (74.29)		-0.029	71.43%	80.57%	-0.091
yes	27 (27.55)	18 (28.57)	9 (25.71)		0.029	28.57%	19.43%	0.091
Organ Metastasis				**0.016**				
no	63 (64.29)	35 (55.56)	28 (80.00)		-0.244	55.56%	49.09%	0.065
yes	35 (35.71)	28 (44.44)	7 (20.00)		0.244	44.44%	50.91%	-0.065

Bold values indicate statistically significant differences (P < 0.05).

### 3.5 Prognostic factor analysis for the IT+RT+CT group and CT+IT group in the first-line and subsequent-line treatments after IPTW

A total of 115.0 effective sample units were retained after IPTW-ATT weighting. The effective sample size of the treatment group (IT+RT+CT) remained 63.0, consistent with its original raw count due to the fixed weight of 1 in the ATT framework. The weighted effective sample size of the control group (CT+IT) was 52.0.

**Figure 5:**
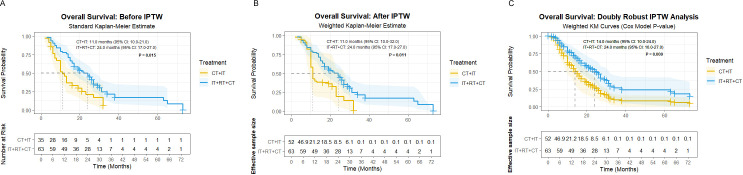
OS of the IT+RT+CT Group and CT+IT Group in the First-line and Subsequent-line Treatments. **(A)** Kaplan-Meier analysis before IPTW. **(B)** conventional IPTW Kaplan-Meier analysis after IPTW. **(C)** Adjusted Kaplan-Meier survival curves generated via the IPTW-ATT doubly robust framework. The number-at-risk table below the curves displays raw patient counts for the IT+RT+CT Group (due to the fixed weight of 1 in the ATT design) and weighted effective sample sizes for the CT+IT Group.

Unadjusted survival analysis showed significant difference between the IT+RT+CT group and the CT+IT group in the First-line and Subsequent-line Treatments, the 1-, 2-year OS were 76.2% versus 46.5% in the IT+RT+CT group, the 1-, 2-year OS were 45.9% versus 21.2% in the CT+IT group, respectively (P = 0.015) (**Figure 5A**). After applying the conventional IPTW Kaplan-Meier analysis, in which the risk sets were directly reweighted using the ATT-specific IPTW weights, the adjusted 1-, 2-year OS were 76.2% versus 46.5% in the IT+RT+CT group, and 39.5% versus 19.4% in the CT+IT group, respectively (P = 0.011) (**Figure 5B**).

Due to extreme weights resulting in a low effective sample size (ESS = 12.02) for the CT+IT group, we applied weight trimming at the 99th percentile. To address residual imbalance in Sex (SMD = 0.11), Age (SMD = 0.24), Tumor Length (SMD = 0.11),TNM stage (SMD = 0.10),Tumor Location (SMD = 0.12), Prior Operation (SMD = 0.14) after weighting, we employed a doubly robust approach by including these covariates in the weighted Cox proportional hazards model (**Table 5**) and the doubly robust IPTW-adjusted survival curves derived from the weighted Cox proportional hazards model (**Figure 5C**). In the First-line and Subsequent-line Treatments, the IT+RT+CT regimen was significantly associated with improved OS compared to the CT+IT regimen (HR: 0.48, 95%CI: 0.27-0.85, P = 0.011) (**Figure 5C**, **Table 5**). Additionally, advanced TNM stage (IVA: HR 7.15, 95%CI: 2.37-21.54, P = 0.000) and larger tumor length (≥5cm: HR 1.93, 95%CI: 1.06-3.54, P = 0.033) were identified as independent predictors of poor survival (**Table 5**).

**Table 5:** Doubly robust Cox proportional hazards model analysis of patients after IPTW.

Variable	Lower_95CI	Upper_95CI	HR	P
First-line and subsequent-line treatments				
CT+IT	1.00 (Reference)			
IT+RT+CT	0.276	0.846	0.483	0.011
Sex				
Female	1.00 (Reference)			
Male	0.716	1.833	1.145	0.571
Age				
<70 years	1.00 (Reference)			
≥70 years	0.572	1.815	1.019	0.950
Tumor length				
<5cm	1.00 (Reference)			
≥5cm	1.056	3.536	1.932	0.033
Unknown	0.155	1.368	0.460	0.163
TNM stage				
I-III	1.00 (Reference)			
IVA	2.372	21.540	7.148	0.000
IVB	0.742	3.638	1.643	0.221
Tumor location				
Middle thoracic	1.00 (Reference)			
Cervical+Upper thoracic	0.409	1.867	0.873	0.727
Lower thoracic+unknown	0.526	1.621	0.923	0.780
Prior operation				
No	1.00 (Reference)			
Yes	0.471	4.282	1.421	0.533

## 4 Discussion

For patients with recurrent and/or metastatic esophageal squamous cancer (ESCC), immunotherapy combined with chemotherapy demonstrated significant survival benefits and become one of the primary treatment (14–17). However, some ESCC patients developed distant lymph node or organ metastases even before disease onset, while others received treatments including radical surgery, concurrent chemoradiotherapy, neoadjuvant chemoradiotherapy, or immunotherapy combined with chemotherapy prior to initial recurrence and metastasis. Therefore, ESCC patients with recurrence and metastasis exhibit significant individual variability and diverse treatment response patterns. For this reason, we conducted a retrospective analysis of the prognosis of ESCC patients at our institution who experienced their first recurrence and metastasis, aiming to evaluate treatment efficacy and provide therapeutic models for this patient subgroup.

Our study found that the median OS were 17.0 months (95%CI:14.0-21.0) and the median PFS was 9.0 months (95%CI:8.0-12.0) (**Figure 1**) for patients with ESCC who experienced first recurrence or metastasis after receiving treatment, which were in line with previous studies (17–19).The therapeutic decision-making for recurrent and/or metastatic ESCC demonstrates considerable heterogeneity, primarily influenced by prior treatment modalities and disease recurrence patterns (5, 8, 9). Based on our study findings, the treatments received by patients prior to the occurrence of recurrence/metastasis (including surgery, radiotherapy, chemotherapy, or immunotherapy) did not significantly impact prognostic survival outcomes. This is because the survival analysis in this study was calculated from the time of first recurrence/metastasis onset rather than the initial diagnosis time, effectively eliminating the time period corresponding to prior treatment phases from the survival evaluation (20, 21). The multivariate analysis demonstrated that pre-recurrence/metastasis stage IVA significantly impacted survival outcomes. The results revealed that patients initially diagnosed with stage IVA disease exhibited poorer survival prognosis after developing recurrence/metastasis compared to those with stage I-III disease upon recurrence or metastasis and Initially diagnosed as stage IVB. This disparity may be attributed to the fact that stage IVA inherently represents locally advanced disease (T4b or N3) with more aggressive biological behavior, whereas stage IVB ESCC patients may derive greater benefit from systemic therapy and targeted treatment approaches.

Notably, the absence of standardized treatment protocols for recurrent disease underscores the need for personalized, multidisciplinary management approaches (5, 9). For patients with initially recurrent/metastatic advanced ESCC, radiotherapy may be a viable therapeutic option when systemic therapies are contraindicated due to economic constraints or poor patient tolerance, particularly in resource-limited settings (22). The present study categorized advanced treatment modalities into four distinct groups: RT alone, RT+CT, RT+CT+IT, and CT+IT. Analysis of these four treatment patterns revealed no significant differences in failure patterns following first-line treatment (detailed in **Table 3**). However, the results demonstrated that the combination of RT+CT+IT significantly improved PFS compared to CT+IT in first-line treatment (**Figure 3**). This finding is consistent with previous research showing that RT+CT+IT combinations improve PFS compared to CT+IT for Stage IVB ESCC with nonregional lymph node metastasis, and that adding radiotherapy to immunotherapy may improve survival outcomes (18, 19). However, this study demonstrated that first-line therapy of RT+CT+IT did not significantly impact overall survival. Multivariate analysis showed that first-line and subsequent-line treatment patterns were independent prognostic factors affecting survival in recurrent/metastatic advanced ESCC. The survival analysis revealed that the combination of RT+CT+IT yielded superior OS compared to CT+IT in first-line and subsequent therapies before and after IPTW (**Figure 5**). This observation can be attributed to the fact that some patients who experienced subsequent recurrence or metastasis went on to receive second-line and further treatments (23–25). In our study, as shown in **Table 1**, an increase of 18 patients was observed in the RT+CT+IT group after progression on first-line therapy. These findings suggest that the selection of optimal treatment strategies tailored to specific recurrence patterns following first-line therapy remains an important area for future investigation.

Our multivariate survival analysis demonstrated no statistically significant differences in OS among the radiotherapy-based treatment modalities (RT+CT, RT alone, and RT+CT+IT groups) in first-line and subsequent-line therapy settings (**Figure 2**). However, there was a trend toward improved OS in patients who received any form of radiotherapy compared to those treated with CT+IT (**Figure 2**). To further analyze the optimal mode of combination with radiotherapy, this study conducted a comparative analysis of the significant survival differences shown in **Figure 2**. Based on our research findings, the RT+CT+IT group demonstrated significantly superior OS compared to the CT+IT group in the first-line and subsequent-line treatments for recurrent/metastatic ESCC. Retrospective analyses suggest that adding radiotherapy to immunochemotherapy may further enhance efficacy in stage IVB ESCC (18, 19). Notably, most existing guideline recommendations for radiotherapy combined with systemic therapy are primarily based on evidence from first-line treatment settings (26). Our real-world data focusing on multi-line treatment for recurrent/metastatic ESCC further supplements the real-world evidence supporting the application of radiotherapy combined with CT+IT in this understudied population. Consistent with existing evidence, the synergy between radiotherapy and immunochemotherapy in ESCC is based on the key mechanisms: radiotherapy enhances systemic antitumor immunity by releasing tumor antigens and reversing the immunosuppressive microenvironment (27). However, this study did not provide detailed analysis regarding the diversity of treatment modalities for recurrent and metastatic ESCC across different recurrence patterns (e.g., distant lymph node metastasis versus organ metastasis, oligometastasis versus widespread metastasis). Larger-scale prospective clinical trials will be required to validate these approaches and establish evidence-based treatment guidelines.

There are several limitations to this study. First, the retrospective nature of the database may result in incomplete or missing data. Comprehensive biomarker data, including PD-L1 expression and baseline tumor burden, was not systematically collected for all enrolled patients, precluding a reliable subgroup analysis to explore their predictive value for patient selection. Second, the database lacks comprehensive and granular data regarding chemotherapy, radiotherapy, and immunotherapy interventions. Specifically, it fails to delineate the distinctions between different chemotherapy protocols (such as platinum-taxane vs. fluorouracil-based regimens) or between various immunotherapy approaches (including anti-PD-1/PD-L1 monotherapy versus combination regimens with chemotherapy).Third, the database does not provide comparative analyses of treatment efficacy or toxicity profiles or quality-of-life implications across sequential lines of therapeutic regimens (e.g., first-line platinum-taxane chemotherapy versus later-line chemo-free regimens, or immune checkpoint inhibitor sequencing strategies). This limitation significantly hinders the ability to evaluate regimen-specific outcomes in advanced disease settings. Furthermore, Detailed documentation and analysis were lacking regarding the prior therapeutic interventions, which encompassed surgical procedures, perioperative treatment strategies (both neoadjuvant and adjuvant approaches), radical radiochemotherapy protocols, and various immunotherapy regimens. The high inter-individual variability in prior interventions, combined with insufficient detailed documentation, resulted in substantial clinical heterogeneity that may have introduced potential bias to our results. Additionally, the small total sample size (N = 156) reduces statistical power for subgroup analysis. To address these issues, we used univariate/multivariate analyses to confirm prior treatment effects, conducted and reported subgroup analyses despite limited power, and applied Inverse Probability of Treatment Weighting (IPTW) to balance baseline characteristics and reduce bias. Therefore, future multi-center, large-scale cohort studies and prospective double-blind randomized clinical trials are needed to further validate the results of this study.

In summary, in terms of tumor response and long-term survival, RT+CT+IT as first-line treatment for recurrent/metastatic ESCC significantly prolonged PFS compared to CT+IT. Furthermore, RT+CT+IT extended OS in First-line and subsequent-line treatments. However, these findings require validation in prospective studies to confirm their clinical significance.

## Data Availability

The raw data supporting the conclusions of this article will be made available by the authors, without undue reservation.
